# Supplementary material for: Pangenomic and Phenotypic Characterization of Colombian Capsicum Germplasm Reveals the Genetic Basis of Fruit Quality Traits
Source: Int J Mol Sci. 2025 Aug 23;26(17):8205. doi: 10.3390/ijms26178205 (PMC12428763; doi:10.3390/ijms26178205)
Supplement: Supplementary file 1 [file ijms-26-08205-s001.zip › Supplementary Materials_MDPI.pdf]

International Journal of *Molecular Sciences*

MDPI

Article

Pangenomic and Phenotypic Characterization of Colombian Capsicum Germplasm Reveals the Genetic Basis of Fruit Quality Traits

Maira A. Vega-Muñoz[1], Felipe López-Hernández[2], Andrés J Cortés[2,3], Federico Roda[5], Esteban Castaño[1], Guillermo Montoya[1,4*], Juan Camilo Henao-Rojas[2,6*]

Supplementary Materials

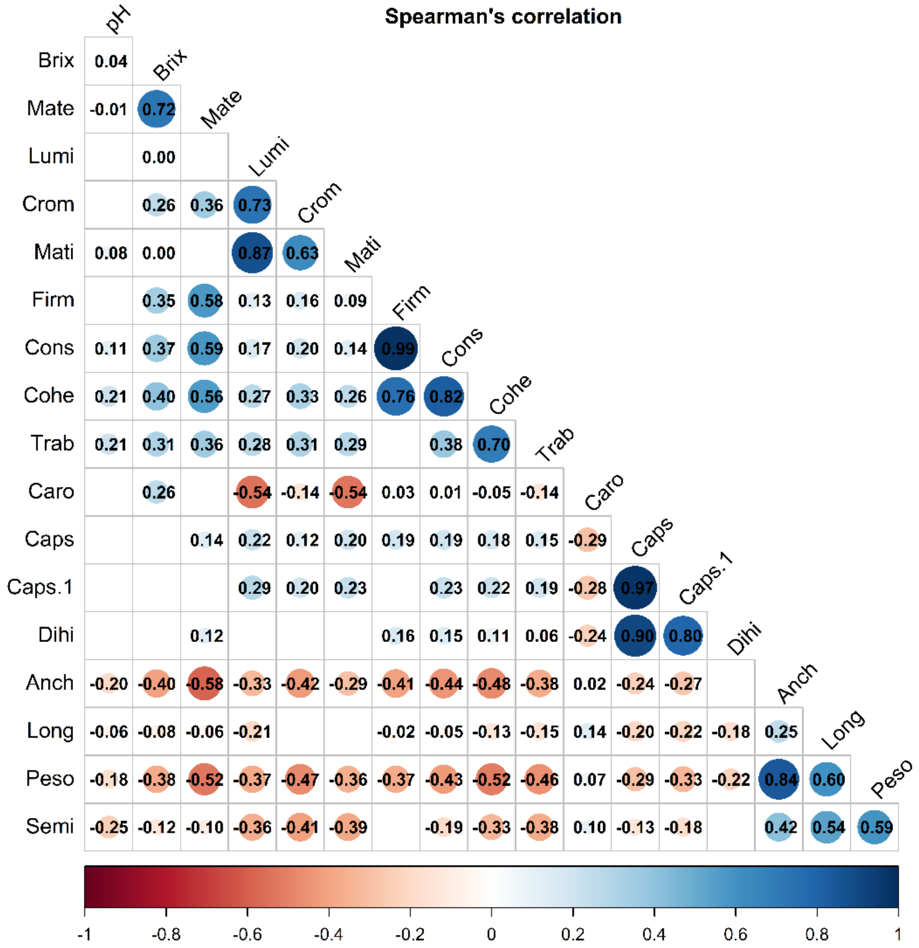

Figure S1: Spearman correlation matrix of phenotypic variables. Brix: soluble solids content; Mate: dry matter; Lumi: luminosity; Crom: chroma; Mati: hue angle; Firm: firmness; Cons: consistency; Cohe: cohesion; Trab: cohesiveness work; Caro: carotenoids; Caps: capsaicinoids; Caps.1: capsaicin; Dihi: dihydrocapsaicin; Anch: fruit width; Long: fruit length; Peso: fruit weight; Semi: seed number per fruit.

International Journal of
Molecular Sciences

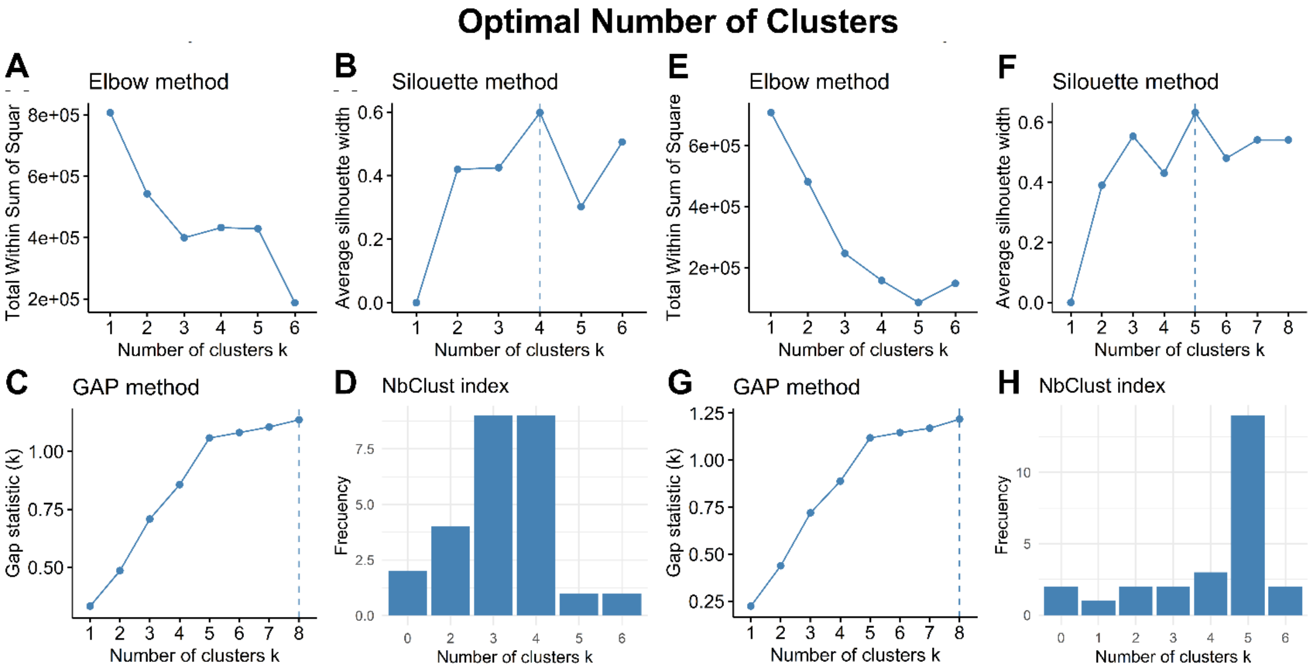

Figure S2. Optimal number of clusters determined by unsupervised clustering methods for both the reference genome and the pangenome. Panels A–D correspond to the reference genome: A) Elbow method; B) Silhouette method; C) GAP statistic method; D) NbClust index. Panels E–H correspond to the pangenome: E) Elbow method; F) Silhouette method; G) GAP statistic method; H) NbClust index.

International Journal of *Molecular Sciences*

MDPI

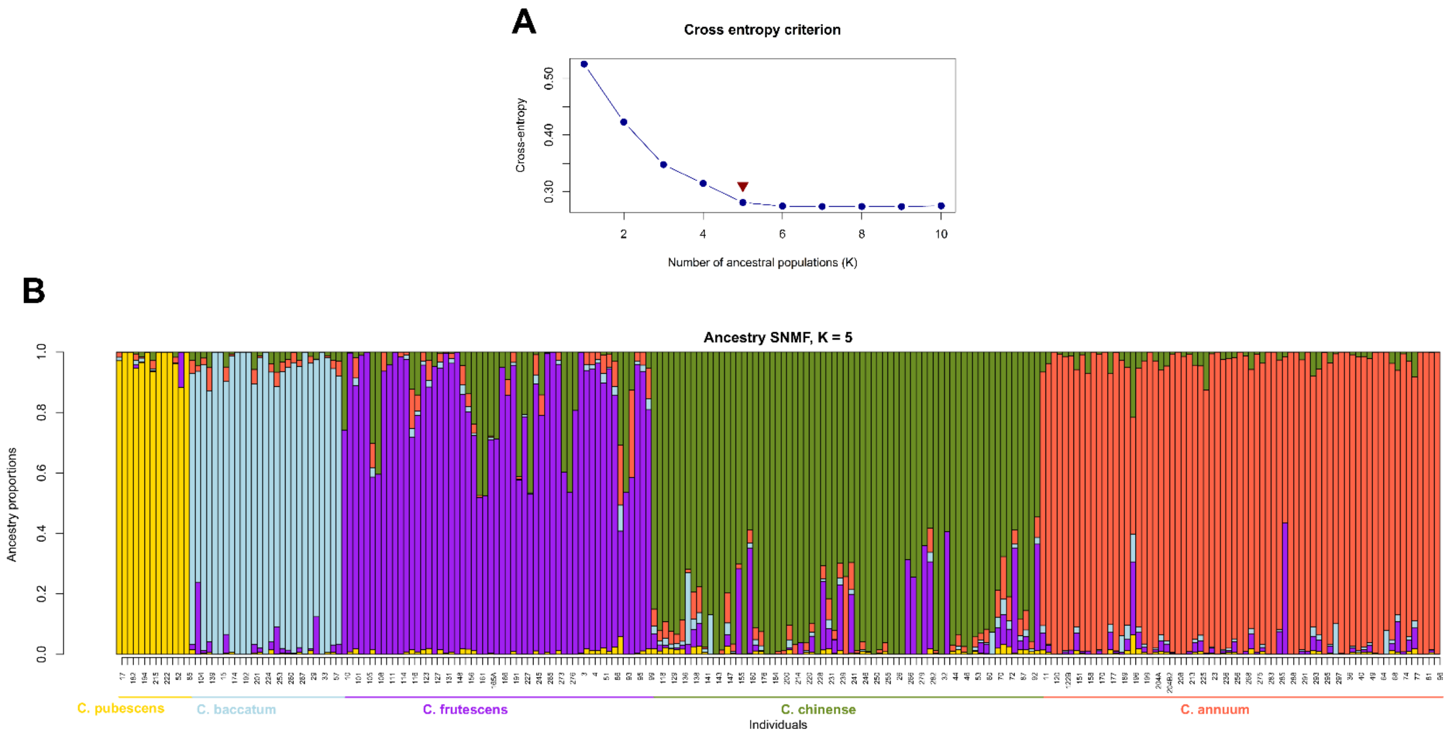

Figure S3. Ancestral population structure of 235 accessions based on the reference genome. A) Optimal number of ancestral populations determined using the cross-entropy criterion. B) Ancestry matrix (admixture proportions) concatenated with species classification, showing the correspondence between genetic structure and taxonomic groups.

International Journal of
Molecular Sciences

MDPI

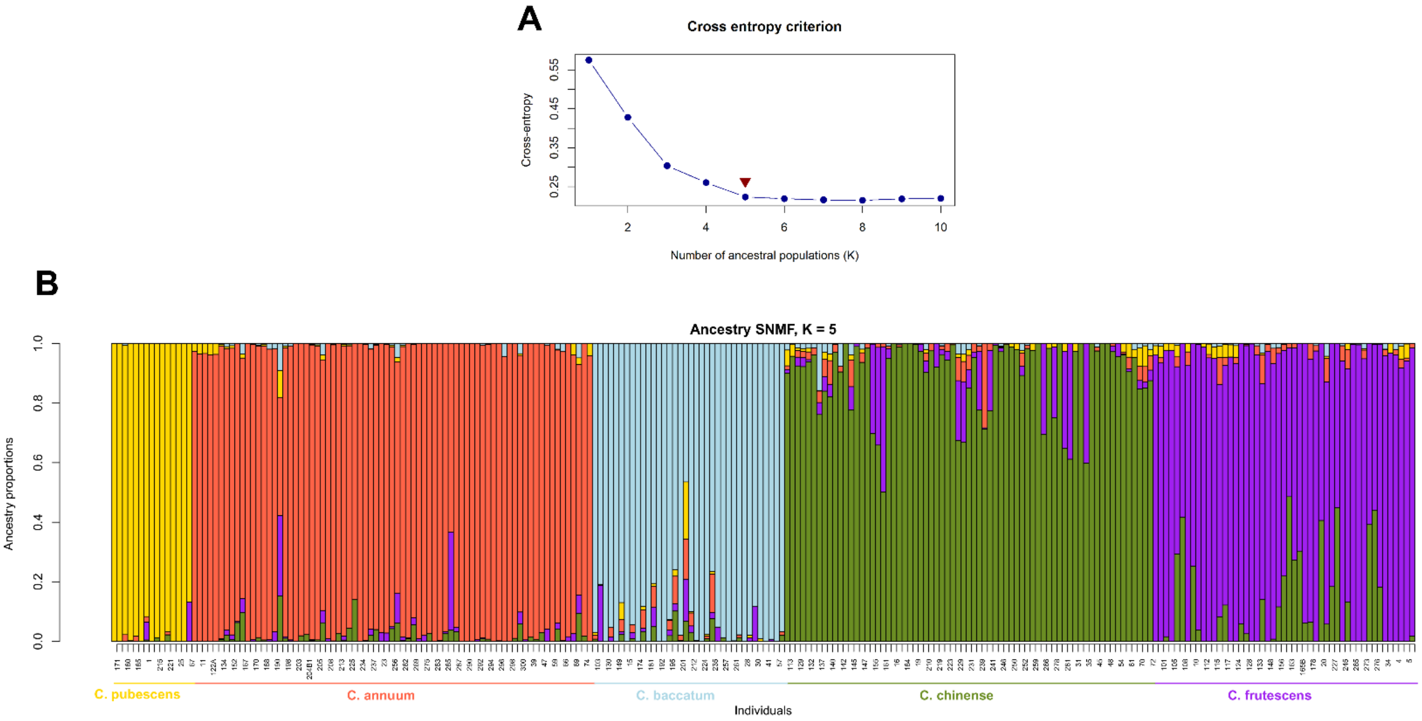

Figure S4. Ancestral population structure of 244 accessions based on the pangenome. A) Optimal number of ancestral populations determined using the cross-entropy criterion. B) Ancestry matrix (admixture proportions) concatenated with species classification, illustrating the relationship between genomic structure and taxonomic identity
